# Obesity-Related Chronic Kidney Disease—The Role of Lipid Metabolism

**DOI:** 10.3390/metabo5040720

**Published:** 2015-12-11

**Authors:** Peter Mount, Matthew Davies, Suet-Wan Choy, Natasha Cook, David Power

**Affiliations:** 1 Department of Nephrology, Austin Health, Heidelberg, Melbourne 3084, Australia; E-Mails: Matthew.Davies@austin.org.au (M.D.); Suet-Wan.Choy@austin.org.au (S-W.C.); Natasha.Cook@austin.org.au (N.C.); David.Power@austin.org.au (D.P.); 2 Department of Medicine, Austin Health, University of Melbourne, Heidelberg 3084, Australia; 3 Institute for Breathing and Sleep, Austin Health, Heidelberg, Melbourne 3084, Australia

**Keywords:** obesity, chronic kidney disease, lipid metabolism, AMP-activated protein kinase, acetyl-CoA carboxylase, high fat diet, podocyte, mesangial cell, proximal tubule

## Abstract

Obesity is an independent risk factor for chronic kidney disease (CKD). The mechanisms linking obesity and CKD include systemic changes such as high blood pressure and hyperglycemia, and intrarenal effects relating to lipid accumulation. Normal lipid metabolism is integral to renal physiology and disturbances of renal lipid and energy metabolism are increasingly being linked with kidney disease. AMP-activated protein kinase (AMPK) and acetyl-CoA carboxylase (ACC) are important regulators of fatty acid oxidation, which is frequently abnormal in the kidney with CKD. A high fat diet reduces renal AMPK activity, thereby contributing to reduced fatty acid oxidation and energy imbalance, and treatments to activate AMPK are beneficial in animal models of obesity-related CKD. Studies have found that the specific cell types affected by excessive lipid accumulation are proximal tubular cells, podocytes, and mesangial cells. Targeting disturbances of renal energy metabolism is a promising approach to addressing the current epidemic of obesity-related kidney disease.

## 1. Introduction

Obesity is an important and independent risk factor for the development and progression of chronic kidney disease (CKD) [1,2]. While it is well known that obesity increases the risk of high blood pressure (hypertension) and diabetes, themselves both major risk factors for CKD [3], studies indicate that hypertension and diabetes only partly explain the association between obesity and CKD, suggesting the involvement of other pathways [4,5]. Excess visceral fat is considered to be the main driving force for all of the various derangements seen in the metabolic syndrome, including the increased risk of CKD [6]. A small subset of morbidly obese individuals develop a specific entity called obesity-related glomerulopathy, characterised by proteinuria with enlargement and scarring of the glomeruli, which are the microscopic filtering units of the kidney [7]. More broadly, however, the mechanisms underlying the strong epidemiological link between excess adiposity and kidney disease are not well understood. In general, both systemic changes and processes within the kidney appear to contribute. Importantly, whilst the observed association between the accumulation of excessive lipids and CKD is well known, there is disagreement as to whether this lipid accumulation is directly toxic, or whether the primary impairment is defective lipid metabolism [8,9,10,11,12].

## 2. Epidemiological Associations between Obesity and CKD

### 2.1. Obesity as a Risk Factor for the Development of Chronic Kidney Disease

There is compelling and consistent epidemiological data that obesity increases the risk of CKD [13]. For example, early observations from the Framingham heart study cohort found increasing levels of body mass index (BMI) conferred a higher risk of CKD, as detected by an elevated serum creatinine level [3]. A systematic review by Wang *et al.*, of the available data up to 2006 concluded that the relative risk of kidney disease was 1.40 with overweight (BMI > 25 kg/m^2^) and 1.83 with obesity (BMI > 30 kg/m^2^) [5]. Notably, this review found that the association of kidney disease with obesity was stronger in women than men (relative risk of 1.92 *vs.* 1.49). Overall, this study estimated that in industrialized countries 13.8% of kidney disease cases in men and 24.9% in women could be related to overweight and obesity [5]. Silverwood *et al.*, found that onset of obesity in earlier adult life predicted subsequent CKD [14]. In this study, being overweight at the ages 26 or 36 years approximately doubled the risk of developing CKD by the age of 60–64 years. These associations were consistent for the development of CKD by several definitions based on measures of either kidney filtration (glomerular filtration rate) or excessive levels of albumin in the urine (albuminuria). Furthermore, the association between obesity and subsequent development of CKD has been extended back to childhood obesity, with an analysis from the 1946 British Birth Cohort study finding that being overweight as a child was associated with a higher risk of CKD, defined by reduced estimated glomerular filtration rate (eGFR), in later life [15]. Obesity also increases the risk of CKD for older individuals. In adults over the age of 65 years, De Boer *et al.*, found that obesity was associated with a higher risk of rapid loss of kidney function [16]. The magnitude of this effect was larger for participants with a baseline eGFR of <60 mL/min/1.73 m^2^. Interestingly, waist circumference, but not BMI or total fat mass, remained a predictor of rapid decline of kidney function after correction for hypertension, diabetes, and inflammation.

### 2.2. Obesity as a Risk Factor for End Stage Kidney Disease

While only a minority of patients with CKD progress to end stage kidney disease (ESKD), defined as either death from kidney failure or the need for dialysis or transplantation, it is this group of patients who bear the greatest disease burden and account for the greatest costs to society. The influence of obesity on ESKD was studied by Hsu *et al.*, who followed 177,570 individuals in Northern California without a prior history of CKD for predictors of progression to ESKD [17]. Over a 25-year follow up period, 842 cases of ESKD were observed. The two strongest risk factors for the development of ESKD were proteinuria and excess weight. For excess weight, the hazard ratios were 4.39 (95% CI, 3.38–5.70) for severe obesity, 3.11 (2.51–3.84) for milder obesity, and 1.65 (1.39–1.97) for overweight *vs.* normal weight. The Norwegian cohort HUNT-1 study of 74,986 adults also observed a relationship between obesity and the risk of ESKD. This study, with a median follow up of 21 years, found an age-adjusted hazard ratio of 1.63 for people who were overweight and 1.94 for those with obesity. This risk for ESKD was further amplified in those with both obesity and elevated blood pressure [18].

### 2.3. Obesity as a Risk Factor for the Progression of Chronic Kidney Disease

Obesity has also been proposed to promote the progression of established CKD of various causes. Bonnet *et al.*, for example, reported that obesity increases the rate of progression of CKD three-fold in patients with IgA nephropathy, a condition characterised by inflammation in the glomeruli that is one of the commonest causes of kidney failure worldwide [19]. Consistent with this, an elevated BMI at the time of IgA nephropathy diagnosis correlated with significantly worse disease at the time of presentation, with more severe hypertension, proteinuria, and more severe renal pathology, as well as worse final outcomes including more severe CKD, dialysis, and death [20]. In addition, being overweight is an independent risk factor for the development of hypertension in patients with IgA nephropathy [19,21]. Othman *et al.*, reported that in a population of non-diabetic CKD patients a higher baseline BMI was associated with faster CKD progression, with the frequency of CKD progression based on eGFR fall per year (>1 mL/min/1.73 m^2^/year) of 79.5% in obese compared to 44.7% in normal weight CKD patients [22]. In contrast, however, a larger study by Brown *et al.*, did not find a relationship between BMI and the rate of progression of CKD in non-diabetic adults [23].

An important question is whether weight loss in obese individuals is able to slow the rate of progression of established CKD. Regarding this, Chagnac *et al.*, has shown that gastroplasty induced major weight loss in individuals with morbid obesity ameliorates obesity related glomerular hyperfiltration, suggesting that this may prevent the development of overt obesity-related glomerulopathy [24]. This supports the data of Praga *et al.*, showing that in obese patients with established proteinuria who were treated by a hypocaloric diet, that there was a significant correlation between body weight loss and reduction in proteinuria [25]. In reviews of the available literature on the effect of weight loss on CKD progression, both Navaneethan *et al.*, and Bolignano *et al.*, found evidence that weight loss reduces proteinuria, thereby suggesting a favourable effect on reducing the progression of CKD [26,27]. Further studies are required, however, to determine whether weight loss can reduce the risk of end stage kidney disease in obese individuals.

## 3. Lipids in Renal Physiology

Lipids are an important constituent of the normal kidney, comprising approximately 3% of its wet weight [8]. More than 50% of the lipids in the kidney are phospholipids, which are the major constituents of cell membranes. Approximately 20% of kidney lipids are triglycerides, and about 10% are free non-esterified free fatty acids (FFAs) [28]. In the kidney, mitochondrial beta-oxidation of FFAs is one of the major sources of ATP production, particularly in the proximal tubule, which has a high energy demand and relatively little glycolytic capacity [29]. This high energy demand of the proximal tubule is explained by its requirement to actively transport large quantities of filtered sodium. Nieth and Schollmeyer found that oxidation of fatty acids accounted for two-thirds of the oxygen consumption of the human kidney and that FFA extraction by the human kidney was linearly dependent on the plasma FFA level over a wide range of concentrations [30]. Whilst most fatty acids in plasma are bound to albumin, it is the unbound FFAs that are taken up by cells via fatty acid translocase (CD36) as well as fatty acid binding proteins [8]. In addition, proximal tubular cells have the capacity to take up albumin bound fatty acids by receptor mediated albumin endocytosis of filtered albumin. Fatty acids taken up by the kidney surplus to its energy requirements can be esterified with glycerol to form triglycerides that are deposited as intracellular lipid droplets. This stored triglyceride is then available to be mobilized for oxidation during the event of energy scarcity.

## 4. Renal Lipid Metabolism and Obesity-Related Chronic Kidney Disease

The observation of fat infiltration in kidney disease was first made over 100 years ago [31]. Building on this, over 30 years ago Moorhead proposed a key role for lipid toxicity in the progression of CKD [32]. This hypothesis was supported by the finding of lipid accumulation in a variety of models of renal disease including the remnant kidney model, acute kidney injury, and diabetic nephropathy [8]. The sites of excess lipid accumulation are predominantly in the proximal convoluted tubule (PCT), podocytes, and mesangial cells [8]. This lipid accumulation has been proposed to be directly toxic, with the principal determinant of the “lipotoxicity” proposed to be excessive intracellular fatty acid content, leading to accumulation of potentially toxic metabolites such as fatty acyl-CoA, diacylgylcerols and ceramides [33]. More direct experimental evidence for the link between obesity and CKD was provided by studies in mice demonstrating the development of kidney disease as a direct consequence of a high fat diet (HFD) [9,10,11,34]. For example, Jiang *et al.*, found that feeding C57Bl/6 mice a HFD for 12 weeks causes glomerulosclerosis and proteinuria, in association with renal accumulation of triglyceride and cholesterol, mediated by the sterol regulatory element-binding protein-1c (SREBP-1c) pathway [11]. The CKD observed with the HFD was associated with increased kidney expression of plasminogen activator inhibitor-1 (PAI-1), vascular endothelial growth factor (VEGF), type IV collagen, and fibronectin [11]. Furthermore, a HFD increases renal lipogenesis with alterations in key biochemical pathways, including reduced activity of AMPK-activated protein kinase (AMPK) and increased expression and activity of both acetyl-CoA carboxylase (ACC) and fatty acid synthase [10].

## 5. AMPK and ACC in Energy and Lipid Metabolism

AMPK is a ubiquitously expressed energy sensing kinase that is activated during energy stress in response to a rise in the AMP/ATP ratio [35]. The response of AMPK is to restore energy balance by stimulating energy generating pathways such as fatty acid oxidation and inhibiting energy consuming pathways such as fatty acid synthesis. Activation of AMPK is widely viewed as a highly promising target for the treatment of problems associated with obesity, metabolic syndrome, and type 2 diabetes. In the kidney, AMPK is abundant and, as we have reviewed in detail [35], various roles have been reported including the regulation of sodium co-transporters and ion channels, as well as the maintenance of podocyte function.

**Figure 1:**
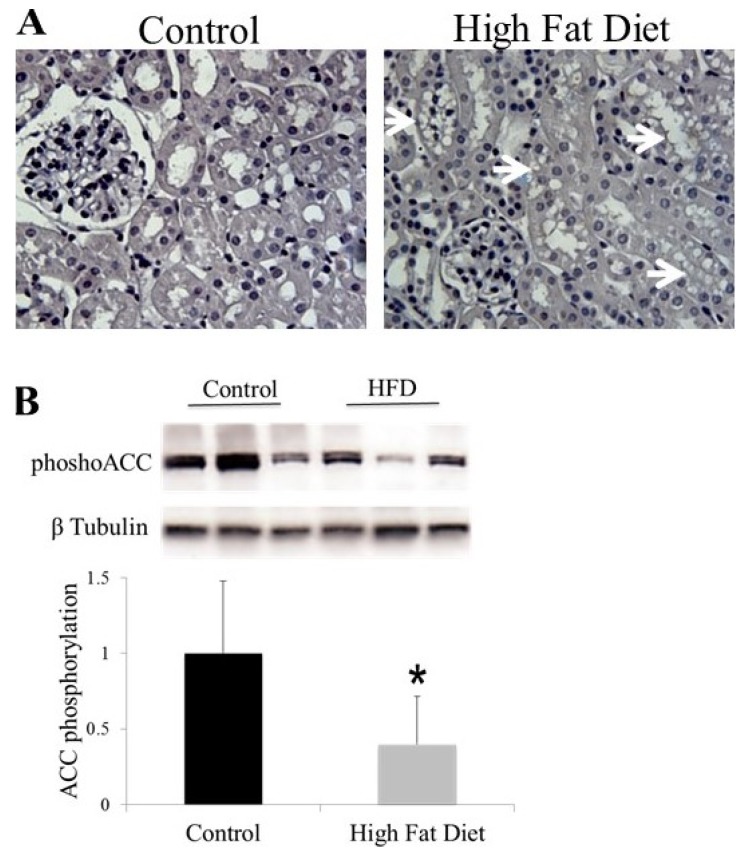
(**A**) C57Bl/6 mice were fed a high fat diet (23% fat by weight) or control diet (5% fat by weight) for 14 weeks. Kidney sections (4 µM) were counterstained with haematoxylin and assessed morphologically. Mice fed a high fat diet developed lipid vacuoles localised to proximal tubular epithelium; (**B**) Reduced ACC phosphorylation (S79/S212) in the renal cortex of mice fed a high fat diet for 14 weeks. Cortical tissue was homogenised and then ACC phosphorylation (S79/S212) was analysed by Western blot with a phosphospecific antibody. *n* = 7. * *p* = 0.008.

The most well-known substrate for AMPK is ACC, which catalyses the carboxylation of acetyl-CoA to malonyl-CoA, the rate limiting step in fatty acid biosynthesis. When AMPK is activated, phosphorylation of ACC causes a fall in malonyl-CoA concentration and, thereby, a reduction in fatty acid synthesis (Figure 1) [36]. Furthermore, the reduced malonyl-CoA concentration increases the activity of carnitine palmitoyl transferase 1 (CPT-1), leading to increased uptake and oxidation of fatty acids by mitochondria. The two isoforms of ACC are ACC1, which is cytoplasmic, and ACC2, which has an N-terminal sequence that localizes it to the mitochondrial membrane. Broadly, ACC1 is more important in the regulation of fatty acid synthesis, whereas ACC2 is more important in the regulation of fatty acid oxidation. Both ACC isoforms are directly phosphorylated by AMPK at Ser^79^ for ACC1 and Ser^212^ for ACC2. We have shown that both ACC1 and ACC2 are expressed in the kidney [37]. The key physiological importance of these ACC phosphorylation sites has been elegantly demonstrated by Fullerton *et al.*, who showed that double serine to alanine knock-in mutations at these sites causes fatty liver disease that is refractory to treatment with the AMPK activating drug metformin [36]. An interesting, related observation is that a single nucleotide polymorphism (SNP) in an intron of the ACC2 gene is associated with development of kidney disease in patients with type 2 diabetes [38].

## 6. AMPK and ACC in Obesity-Related Kidney CKD

A consistent finding in the kidneys of mice on a HFD is increased ACC activity, which appears to be a direct consequence of reduced ACC (Figure 1) phosphorylation as a consequence of reduced AMPK activity in the kidney [10,34]. This then promotes lipid accumulation within kidney cells, with potential to produce lipid toxicity. The specific mechanisms responsible for the reduced AMPK activity in the kidney with HFD are not clearly established but it has been proposed to be explained by energy excess leading to a reduced AMP/ATP ratio [34]. Another proposed contributor to reduced AMPK activity in the kidney with obesity is reduced serum adiponectin. Adiponectin has been shown to activate AMPK in podocytes and tubular cells via the ADIPOR1 receptor [39,40].

Evidence that pharmacological activation of AMPK protects from the development of obesity-related CKD was seen with the AMPK activator 5-Aminoimidazole-4-carboxamide ribonucleotide (AICAR), which, in a short term study, reduced renal hypertrophy and markers of oxidative stress (urine H_2_O_2_) and inflammation (MCP-1) occurring with a HFD [34]. Moreover, in a longer term study, administration of AICAR for 14 weeks prevented the clinical and structural renal consequences of a HFD [41]. Activation of AMPK by the diabetes drug metformin has also been reported to reduce HFD induced renal injury [42]. In the db/db model of obesity and type 2 diabetes the AMPK and Sirtuin 1 activator resveratrol, found in red wine, prevented lipid accumulation and the development of CKD, implicating both molecules as well as downstream effectors such as peroxisome proliferator-activated receptor (PPAR)γ co-activator 1α (PGC-1α), PPARα–oestrogen-related receptor (ERR)-1, and α–sterol regulatory element-binding protein 1 (SREBP1) [43].

## 7. Lipid Accumulation and Toxicity in Proximal Tubular Cells

**Figure 2:**
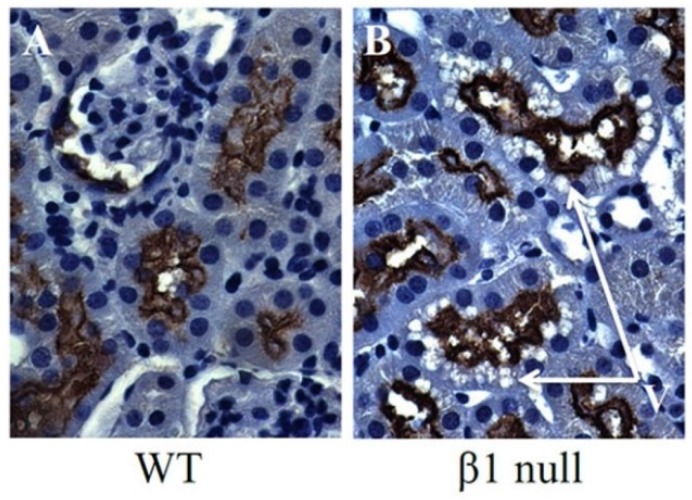
Immunohistochemistry from AMPK knockout mice deficient for the AMPK-β1 subunit (β1 null). Tubular vacuolation, it is noted, co-localises with staining for the proximal tubular marker megalin. V = vacuoles.

The primary role for the cells of the proximal tubule is the active reabsorption of a large quantity of filtered sodium, by a process that requires a large amount of energy that is predominantly derived from fatty acid β-oxidation. Our laboratory and others have observed that HFD feeding in mice causes accumulation of lipid vacuoles in proximal tubular cells (Figure 1A) [34]. Furthermore, this lipid accumulation is associated with reduced activity of AMPK in the kidney, which then leads to reduced phosphorylation and increased activity of ACC (Figure 1B). Interestingly, we have also observed a similar pattern of proximal tubular lipid vacuolation in mice that are deficient for the AMPK-β1 subunit (Figure 2), providing further evidence that reduced AMPK in the kidney results in proximal tubular cell lipid accumulation.

The importance of renal tubular lipid accumulation, related to impaired fatty acid oxidation, in the pathogenesis of CKD has recently been demonstrated by Kang *et al.*, who showed that this is a prominent feature of renal fibrosis in human kidney disease [12]. By a genome-wide transcriptome analysis these investigators found lower expression of key enzymes and regulators of fatty acid oxidation, in both human CKD biopsies and mouse models with tubulointerstitial fibrosis [12]. This is important because tubulointerstitial fibrosis is the final common pathway of widespread scarring for all kidney diseases leading to chronic renal failure [44]. Significantly, in the mouse models, reduced expression of fatty acid oxidation genes preceded the development of fibrosis, suggestive of a causal relationship [12]. Furthermore, in tubular epithelial cells they found that inhibition of fatty acid oxidation led to intracellular lipid deposition, cellular dedifferentiation, and cell death. Importantly, these investigators also found that restoring fatty acid metabolism by genetic or pharmacological methods protected mice from tubulointerstitial fibrosis [12].

An important mechanistic question regarding the observed association between renal tubular lipid accumulation and CKD, is whether the accumulated lipid is directly pathogenic or whether the primary defect is energy deficiency related to an impaired fatty acid oxidation. To address this, Kang *et al.* generated a mouse model with kidney-specific tetracycline inducible overexpression of fatty acid translocase (CD36). After 20 weeks these mice developed marked lipid accumulation, but the effect on fibrosis was minimal, with only minor increases in the expression of *Col1A* (type 1 collagen) and *Acta2* (smooth muscle actin) seen [12]. Furthermore, the CD36 renal transgenic mice did not show increased susceptibility to diabetic kidney injury or folic acid-induced fibrosis, although surprisingly these mice were not tested in a HFD induced CKD model. From these findings, Kang *et al.*, proposed that reduced fatty acid oxidation could be the major factor for fibrosis development, with lipid accumulation occurring as a secondary consequence. A remaining question, however, is that whilst this may explain the association of various causes of CKD with lipid accumulation, it does not seem to explain the association between obesity and CKD, that is seen in both humans and animal models. Furthermore, these data should be interpreted in the context of cell culture studies, in which exposure of cells to increased concentrations of free fatty acids appears to be pathogenic [45,46,47].

It has also been shown that kidneys from mouse models of progressive, albuminuric kidney disease exhibit increased proximal tubular fatty acids and long-chain fatty acyl-CoAs, as well as more caspase-2-dependent lipoapoptosis [46]. Interestingly, this lipotoxic effect was shown to be mediated by an interaction between long chain acyl-CoAs and the Na^+^/H^+^ exchanger NHE1 [46]. Increased susceptibility to lipid accumulation is a particular characteristic of proximal tubule cells, with fatty acids entering from both the basolateral (blood) and the luminal (urine) sides [8]. The luminal fatty acid uptake and accumulation by proximal tubular cells has been linked with albumin reabsorption in proteinuric states [8,46], potentially contributing to the known correlation between glomerular diseases and tubulointerstitial fibrosis.

## 8. Lipid Accumulation and Toxicity in Podocytes

Podocytes are the cells in the Bowman’s capsule in the kidneys that wrap around the capillaries of the glomerulus. They have a crucial role in the normal filtering process of the kidney. Importantly, podocytes are the cell type primarily affected in obesity-related glomerulopathy, which is characterised by a pattern of focal glomerulosclerosis and progressive renal failure [48]. Furthermore, in animal models a HFD leads to accumulation of glomerular lipid and podocyte loss [11]. Significantly, podocytes have been demonstrated to be directly susceptible to toxicity mediated by saturated FFAs, such as palmitic acid, which induce endoplasmic reticulum (ER) stress and podocyte death [45]. This effect was abrogated by stimulation of fatty acid oxidation by AMPK activators, with the benefit of AMPK activation being mediated by phosphorylation of both the ACC1 and ACC2 isoforms [45]. In contrast, inhibition of fatty acid oxidation by the CPT-1 inhibitor etomoxir increased the toxic effect of palmitic acid on podocytes [45]. Another important observation is that AMPK activation reduced albuminuria and restored podocyte structure and function in adiponectin deficient mice [39]. Interestingly, several podocyte genes that have recently been associated with human kidney disease, such as ApoL1 and PLA2R, are involved in lipid metabolism [49]. It has also been proposed that FFA accumulation in podocytes might explain the association between polymorphisms of the ACC2 gene and diabetic nephropathy [38].

## 9. Lipid Accumulation and Toxicity in Mesangial Cells

Intraglomerular mesangial cells are specialized pericytes located amongst the capillary loops of the glomerulus. The functions of mesangial cells include structural support of capillary loops, regulation of capillary flow and glomerular filtration, phagocytosis, and regulation of the mesangial matrix [50]. Mesangial cells take up cholesterol esters via low density lipoprotein (LDL) receptors, and modified LDL and long-chain fatty acids via scavenger receptors [51]. Mesangial cells also express lipoprotein lipase, which has been shown to be important for mesangial cell accumulation of triglycerides resulting from exposure to very low density lipoprotein (VLDL) [52]. In the setting of inflammation, mesangial cells become lipid laden, taking on a foam cell appearance. The mechanism appears to be a cytokine mediated failure to appropriately physiologically downregulate the LDL-receptor [53]. Compared to normal mesangial cells, lipid laden foam cells have abnormal function, thereby disturbing normal glomerular regulation. For example, Berfield *et al.*, found that insulin-like growth factor induced mesangial cell lipid accumulation led to impaired contractile and migratory responses [54]. Studies have shown that activation of AMPK, by resveratrol or the PPARα agonist fenofibrate for example, can reduce lipid accumulation and restore healthy mesangial cell function in diabetes and hyperglycaemia [43,55].

## 10. Conclusions

**Figure 3:**
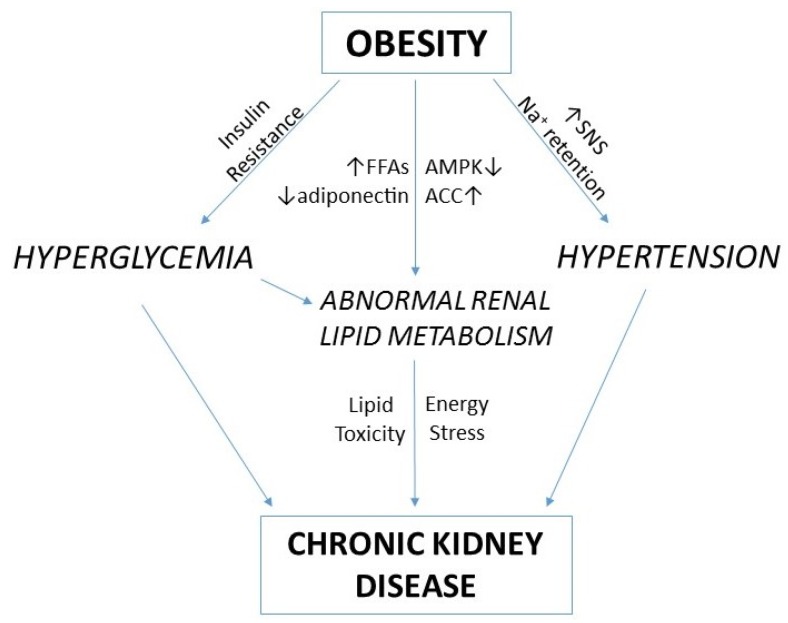
A proposed pathogenesis of obesity-related chronic kidney disease. Obesity leads to CKD through both systemic effects such as hypertension and hyperglycemia, and intrarenal effects associated with impaired lipid metabolism. Sodium retention and activation of the sympathetic nervous system (SNS) result in hypertension, whilst insulin resistance causes hyperglycemia. Energy excess associated with increased free fatty acids (FFAs) and reduced adiponectin, reduces the activity of AMP-activated protein kinase (AMPK), thereby increasing the activity of acetyl-CoA carboxylase (ACC). The resulting reduction in fatty acid oxidation and increase in fatty acid synthesis, promotes a situation of lipid toxicity and energy stress which further contributes to the development of CKD.

Obesity is established as an important and independent risk factor for CKD. The mechanisms underlying this association involve systemic effects such as hypertension, hyperglycemia, and dyslipidemia. In addition, intrarenal effects such as impaired fatty acid oxidation and lipid accumulation may further contribute to CKD pathogenesis (Figure 3). Current therapeutic options for preventing and treating CKD are basically limited to systemic interventions to reduce blood pressure. As evidence continues to accumulate demonstrating the link between disturbances of renal energy metabolism and kidney disease, this should open up new therapeutic avenues that target renal metabolism. Strategies that target fatty acid oxidation, for example via AMPK or PPARα, appear particularly promising with the potential to increase both cellular energy availability and to correct the adverse effects that might result from lipid accumulation.
